# Losing self control

**DOI:** 10.7554/eLife.21404

**Published:** 2016-10-20

**Authors:** Luke Miller, Alessandro Farnè

**Affiliations:** ImpAct Laboratory, Lyon Neuroscience Research Centre, Lyon, France; ImpAct Laboratory, Lyon Neuroscience Research Centre, Lyon, Francealessandro.farne@inserm.fr

**Keywords:** body ownership, primary motor cortex, magnetic transcranical stimulation, Human

## Abstract

Our brain is less able to move one of our hands if an illusion makes us feel like the hand does not belong to us.

**Related research article** della Gatta F, Garbarini F, Puglisi G, Leonetti A, Berti A, Borroni P. 2016. Decreased motor cortex excitability mirrors own hand disembodiment during the rubber hand illusion. *eLife* **5**:e14972. doi: 10.7554/eLife.14972

We are all familiar with the feeling that our body is our own and that we can control it. Indeed, most of us have probably never given more than a passing thought to the complexities that underlie our awareness of the body and self. However, these complexities are revealed in dramatic fashion by forms of brain damage that alter the feeling of ownership of body parts. Following damage to key regions of the frontal and parietal cortex, patients may deny that their paralyzed hand is their own, or may claim ownership of someone else's hand instead (Vallar and Ronchi, 2009).

There is considerable evidence that motor actions, such as reaching to grab an object, provide the glue that binds the body with the self (Jeannerod, 2006). Various body-based illusions, such as the rubber hand illusion, have also implicated sensory information processing in the construction and maintenance of the bodily self (Ehrsson et al., 2004; Blanke et al., 2015). Now, in eLife, Francesca Garbarini and colleagues – including Francesco della Gatta as first author – report that even the motor system is regulated by levels of body ownership (della Gatta et al., 2016).

In the rubber hand illusion, an experimenter typically strokes a participant’s hand that is hidden from view at the same time as stroking a prosthetic hand (usually made of rubber) placed in front of the participant (Botvinick and Cohen, 1998). This leads to participants reporting that they feel as if the tactile sensations they are experiencing originate in the prosthetic hand. Furthermore, they report feeling that the prosthetic hand has become incorporated into their body, a phenomenon called embodiment. One of the most intriguing consequences of this illusion is that participants report that their own hand feels less vivid, as if it has become ‘disembodied’ (Longo et al., 2008).

Beyond such subjective reports, disembodiment of the hand during the illusion is accompanied with a reduction in skin temperature (Moseley et al., 2008). It is possible that the illusion may also affect how the hand is represented in the motor system. Now della Gatta et al. – who are based at the University of Turin and the University of Milan – have directly investigated the link between disembodiment and an individual’s motor capabilities in a simple, yet elegant experiment.

Transcranial magnetic stimulation (TMS) is a non-invasive procedure that uses magnetic fields to stimulate activity in specific regions of the brain. A single pulse of TMS to a specific part of the primary motor cortex leads to a measurable twitch in the muscles of the hand on the opposite side of the body (Figure 1, left panel). The size of this twitch, which is called a motor-evoked potential, increases with the level of electrical excitability of motor neurons in the cortico-spinal tract connected to the muscle.

**Figure 1.**
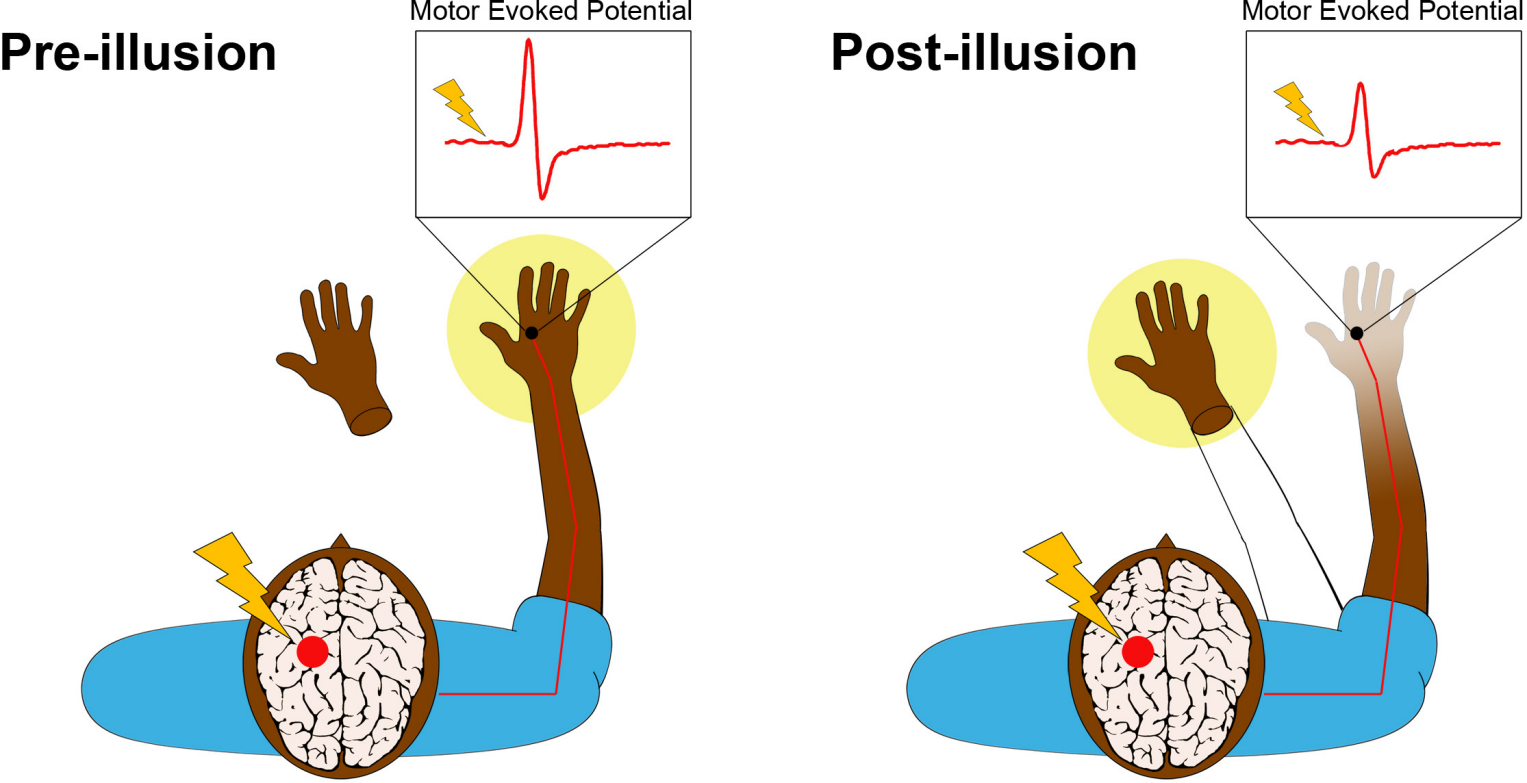
The rubber hand illusion alters the way the hand is represented in the motor cortex. Before the illusion starts (left panel), the participant feels that their right hand, which is hidden from view, belongs to their body (yellow spotlight) and that the prosthetic hand does not belong. della Gatta et al. applied a single pulse of transcranial magnetic stimulation (lightning bolt) to the region of the left primary motor cortex that controls the right hand (red circle). This causes an electrical pulse to travel down the corresponding motor nerves in the right arm (red line) to the target muscle in the right hand, where an electrode (black circle) records a rapid burst of electrical activity called a motor-evoked potential (i.e., muscle twitch). This potential is illustrated in the inset above the hand. After the illusion (right panel), the prosthetic hand has become embodied, meaning that the participant feels like it is now part of their body (yellow spotlight and outline of an arm). The real right hand, conversely, feels less vivid to the participant. Furthermore, the size of the motor-evoked potential has significantly decreased, providing an objective measure of limb disembodiment.

Leveraging this relationship, della Gatta et al. used single-pulse TMS to trigger motor-evoked potentials in the right hands of human volunteers before, during and after the induction of the rubber hand illusion in the right hand. The team found that the amplitude of the potentials decreased significantly during the illusion (Figure 1, right panel). Crucially, this decrease was only observed when the real hand and the rubber hand were stroked at the same time. The volunteers felt that the rubber hands became part of their bodies while their real right hands became disembodied. Furthermore, the size of the motor-evoked potentials continued to decrease over time, presumably tracking increasing levels of disembodiment (which was not measured in this study). There was no change in the size of motor-evoked potentials in the left hand, which was not targeted during the illusion.

Our experience of embodiment and body ownership is intimately related to our ability to act on the world around us (de Vignemont, 2011; Bolognini et al., 2016). For example, tools get embodied into the representations of the sensory and motor (sensorimotor) system in the brain (Miller et al., 2014; Martel et al., 2016) and an illusion of limb amputation in virtual reality regulates the excitability of motor neurons (Kilteni et al., 2016). Our actions are also tied to the bodily self, as volunteers respond to pictures of their own body parts faster than they respond to pictures of body parts belonging to someone else (Frassinetti et al., 2011).

The work of della Gatta et al. represents a major advance in our understanding of our sense of body ownership by demonstrating that the state of the sensorimotor system is intimately tied to a limb’s current state of embodiment. Future work should aim to track fluctuations in embodiment and the motor system in real-time, thus providing a richer understanding of the embodied motor self.
